# Conservation and sustainable use of medicinal plants: problems, progress, and prospects

**DOI:** 10.1186/s13020-016-0108-7

**Published:** 2016-07-30

**Authors:** Shi-Lin Chen, Hua Yu, Hong-Mei Luo, Qiong Wu, Chun-Fang Li, André Steinmetz

**Affiliations:** 1 Institute of Chinese Materia Medica, China Academy of Chinese Medical Sciences, Beijing, 100700 China; 2 Institute of Medicinal Plant Development, Chinese Academy of Medical Sciences, Beijing, 100193 China; 3 Shandong Center of Crop Germplasm Resources, Jinan, 250100 China; 4 School of Pharmacy, Guilin Medical University, Guilin, 541004 China; 5 Plant Molecular Biology Laboratory, Centre de Recherche Public-Sante´, 1526 Luxembourg, Luxembourg

## Abstract

Medicinal plants are globally valuable sources of herbal products, and they are disappearing at a high speed. This article reviews global trends, developments and prospects for the strategies and methodologies concerning the conservation and sustainable use of medicinal plant resources to provide a reliable reference for the conservation and sustainable use of medicinal plants. We emphasized that both conservation strategies (e.g. in situ and ex situ conservation and cultivation practices) and resource management (e.g. good agricultural practices and sustainable use solutions) should be adequately taken into account for the sustainable use of medicinal plant resources. We recommend that biotechnical approaches (e.g. tissue culture, micropropagation, synthetic seed technology, and molecular marker-based approaches) should be applied to improve yield and modify the potency of medicinal plants.

## Background

Medicinal plants are globally valuable sources of new drugs [1–4]. There are over 1300 medicinal plants used in Europe, of which 90 % are harvested from wild resources; in the United States, about 118 of the top 150 prescription drugs are based on natural sources [5]. Furthermore, up to 80 % of people in developing countries are totally dependent on herbal drugs for their primary healthcare, and over 25 % of prescribed medicines in developed countries are derived from wild plant species [4]. With the increasing demand for herbal drugs, natural health products, and secondary metabolites of medicinal plants, the use of medicinal plants is growing rapidly throughout the world [1, 6].

A highly conservative estimate states that the current loss of plant species is between 100 and 1000 times higher than the expected natural extinction rate and that the Earth is losing at least one potential major drug every 2 years [7]. According to the International Union for Conservation of Nature and the World Wildlife Fund, there are between 50,000 and 80,000 flowering plant species used for medicinal purposes worldwide. Among these, about 15,000 species are threatened with extinction from overharvesting and habitat destruction [8] and 20 % of their wild resources have already been nearly exhausted with the increasing human population and plant consumption [9]. Although this threat has been known for decades, the accelerated loss of species and habitat destruction worldwide has increased the risk of extinction of medicinal plants, especially in China [1, 10], India [10, 11], Kenya [11], Nepal [11], Tanzania [12] and Uganda [12].

The conservation and sustainable use of medicinal plants have been studied extensively [13, 14]. Various sets of recommendations have been compiled regarding their conservation, including the establishment of systems for species inventorying and status monitoring, and the need for coordinated conservation practices based on both in situ and ex situ strategies [4]. For medicinal plants with increasingly limited supplies, sustainable use of wild resources can be an effective conservation alternative. In China and South Africa, the situation is particularly critical because of the high demands of large populations.

This article reviews global trends, developments and prospects of the strategies and methodologies concerning the conservation and sustainable use of medicinal plant resources.

## Review

### Literature search

**Fig. 1:**
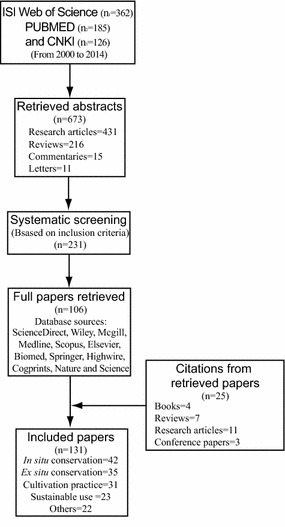
Diagram for literature selection

We conducted systematic literature searches in the ISI Web of Science, PubMed and CNKI databases using search terms (e.g. “medicinal plant”, “herbal drug”, “herbal medicine”, “aromatic plant”, and “Chinese medicine”) and keyword combinations (e.g. “in situ conservation”, “ex situ conservation”, “sustainable use”, and “cultivation practice”) to retrieve the abstracts of relevant articles (Fig. 1). The literature search spanned the period from January 2000 to December 2014, and the languages were limited to English and Chinese. We carefully reviewed all retrieved abstracts to find articles that were suitable according to the inclusion criteria (Table 1). For literature focusing on the conservation and/or sustainable use of medicinal plants, we retrieved full articles from full-text databases (ScienceDirect, Wiley, Biomed, Springer, Medline, Scopus, Elsevier, Highwire, Mcgill, Cogprints, Emedicine, Nature and Science online). From the initial search, a total of 673 abstracts were collected, including research articles, reviews, commentaries and letters. After systematic screening, 231 met the inclusion criteria. From these, full copies of 106 articles were retrieved for further evaluation. In addition, we retrieved 25 non-indexed but relevant citations from the reference lists of retrieved articles to supplement the above searches and provide a complete literature retrieval.

**Table 1:** The inclusion and exclusion criteria for study selection

Subjects	Criteria description
Inclusion criteria	The subject is about medicinal plants
	The purpose is to conserve or sustainably use
	It has a detailed description of the strategy of conservation, sustainable use or resource management
	Consistent to the three items simultaneously
Exclusion criteria	The subject is not related to medicinal plants (e.g. animals or mineral substances)
	The purpose is not for conservation or sustainable use of medicinal plants (e.g. herb extraction, drug discovery, chemical constituent or pharmacological property)
	It has no description of the strategy of conservation, sustainable use or resource management of medicinal plants

### Diversity of medicinal plants used worldwide

**Fig. 2:**
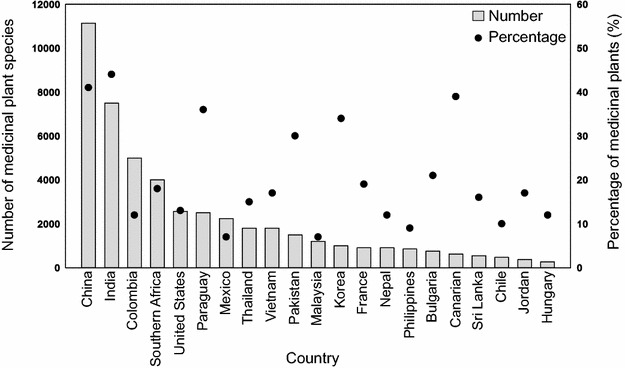
Number and percentage of medicinal plant species in different countries. The *light bars* indicate the number of medicinal plant species, and the *dark dots* indicate the percentage of medicinal plants compared with the total number of plant species. Data sources from Rafieian-Kopaei [16], Hamilton [17], Marcy et al. [18], and Srujana et al. [19]

More than one-tenth of plant species are used in drugs and health products, with more than 50,000 species being used. However, the distribution of medicinal plants is not uniform across the world [15, 16]. For example, China and India have the highest numbers of medicinal plants used, with 11,146 and 7500 species, respectively, followed by Colombia, South Africa, the United States, and another 16 countries with percentages of medicinal plants ranging from 7 % in Malaysia to 44 % in India versus their total numbers of plant species [16–19] (Fig. 2). Certain plant families not only have higher numbers of medicinal plants, but also have higher proportions of threatened species than others [15]. Only a portion of medicinal plants that suffer from genetic erosion and resource destruction have been listed as threatened [20, 21].

### Factors related to species rarity of medicinal plants

**Fig. 3:**
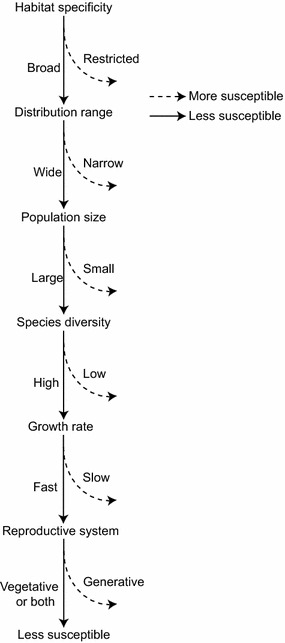
Factors contributing to the susceptibility or resilience of medicinal plants in response to collection pressure. Factors include distribution range, habitat specificity, population size, species diversity, growth rate, and reproductive system. The *dark line* indicates less susceptible characteristics of medicinal plants, while the *dashed line* indicates more susceptible characteristics contributing to the rarity of medicinal plants

Species rarity is used to assess the extinction risk of medicinal plants, and to identify those species most at risk of extinction, prior to commencement of conservation efforts [22]. It is necessary to determine how rare each species is and in which ways rare species differ from one another. Not all medicinal plants are affected in the same way by harvesting pressures [23, 24]. Overexploitation, indiscriminate collection, uncontrolled deforestation, and habitat destruction all affect species rarity, but are insufficient to explain individual species susceptibility or resilience to harvest pressure. Multiple biological characters correlate with extinction risk, such as habitat specificity, distribution range, population size, species diversity, growth rate, and reproductive system (Table 2; Fig. 3).

**Table 2:** Summary of original investigations into the conservation and sustainable use of medicinal plants

Sources	Objectives	Features and study design	Findings and conclusions
Andel and Havinga [24]	249 medicinal plants in Suriname	Carried out a market survey to look for signs of overharvesting by analyzing the market, harvesters and post-harvest survival of the medicinal plants	Less than half of the medicinal plants were harvested exclusively from the wild, and leaves were the main products. Most medicinal plants were harvested from secondary forest or man-made vegetation. It didn’t invariably lead to the resource decline or species loss
Semwal et al. [26]	Ten rare and endangered medicinal plants	Based on the density, occurrence habitats and pressure level to evaluate the distribution pattern, population structure and conservation status of medicinal plants	It grouped the medicinal plants into restricted distribution with high pressure and well distributed with low pressure, providing insights for the conservation and management strategies of medicinal plants
Long et al. [32]	*Amorphophallus*, *Paris*, *Musella lasiocarpa*, and *Camellia sinensis*	Dealt with strategies for agrobiodiversity conservation and promotion based on studies of the medicinal plants in the Yunnan Province of China	Strategies (e.g. in situ and ex situ conservation, as well as the promotion and conservation of agrobiodiversity through sustainable uses) should be adopted to conserve and promote agrobiodiversity
Strandby and Olsen [41]	*Abies guatemalensis*	From December 2004 to August 2007 conducted a nation-wide survey with plantation owners (n = 26), retailers (n = 67) and urban consumers (n = 993)	It emphasized the importance of increasing legal supplies through decreasing plantation production costs and involving local communities in managing in situ resources
Yu et al. [44]	*Rheum tanguticum*	Employed a GIS-based program TCMGIS-II to integrate geographic, climate and soil type databases of China to predict the potential distribution of *R. tanguticum*	It found the potential habitats sharing similar ecological factors with native habitats appropriate for *R. tanguticum* growth. It is useful in the conservation planning and regional management of medicinal plants
Yuan et al. [47]	*Scutellaria baicalensis*	Estimated the genetic diversity and structure of 28 wild and 22 cultivated populations of *S. baicalensis* using three polymorphic chloroplast fragments	The conservation-by-cultivation is effective in protecting genetic resources, while the wild resources still need to be protected in situ. The evolutionary consequences of extensive seed exchange should be monitored carefully
Maunder et al. [49]	27 threatened medicinal plants	Surveyed 119 botanic gardens in 29 European countries, and 25 botanic gardens in 14 countries undertaking 51 conservation projects	Most medicinal plants were in a small number of collections and out of the range countries, without being included in any specific conservation project. Botanic garden collections were skewed towards ornamental species, and did not fully reflect conservation priorities

*GIS* geography information systems, *TCMGIS-II* the second version of GIS based program for the distribution prediction of traditional Chinese medicine

### Conservation strategies

**Fig. 4:**
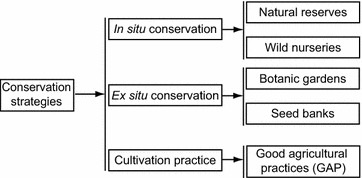
Diagram of methodological systems involved in the conservation of medicinal plants

Medicinal plant resources are being harvested in increasing volumes, largely from wild populations. Indeed, demand for wild resources has increased by 8–15 % per year in Europe, North America, and Asia in recent decades [8, 9]. There is a threshold below which species reproductive capacity becomes irreversibly reduced [25, 26]. Various sets of recommendations relating to the conservation of medicinal plants have been developed, such as providing both in situ and ex situ conservation (Table 2) [15, 27]. Natural reserves and wild nurseries are typical examples to retain the medical efficacy of plants in their natural habitats, while botanic gardens and seed banks are important paradigms for ex situ conservation and future replanting [28, 29] (Fig. 4). The geographic distribution and biological characteristics of medicinal plants must be known to guide conservation activities, e.g. to assess whether species conservation should take place in nature or in a nursery.

#### In situ conservation

Most medicinal plants are endemic species, and their medicinal properties are mainly because of the presence of secondary metabolites that respond to stimuli in natural environments, and that may not be expressed under culture conditions [22, 29]. In situ conservation of whole communities allows us to protect indigenous plants and maintain natural communities, along with their intricate network of relationships [30]. Additionally, in situ conservation increases the amount of diversity that can be conserved [31], and strengthens the link between resource conservation and sustainable use [32]. In situ conservation efforts worldwide have focused on establishing protected areas and taking an approach that is ecosystem-oriented, rather than species-oriented [33]. Successful in situ conservation depends on rules, regulations, and potential compliance of medicinal plants within growth habitats [25, 34].

##### Natural reserves

The degradation and destruction of habitats is a major cause of the loss of medicinal plant resources [35]. Natural reserves are protected areas of important wild resources created to preserve and restore biodiversity [36, 37]. Around the world, more than 12,700 protected areas have been established, accounting for 13.2 million km^2^, or 8.81 % of the Earth’s land surface [38]. Conserving medicinal plants by protecting key natural habitats requires assessing the contributions and ecosystem functions of individual habitats [39].

##### Wild nurseries

It is impossible to designate every natural wild plant habitat as a protected area, owing to cost considerations and competing land uses [25, 40]. A wild nursery is established for species-oriented cultivating and domesticating of endangered medicinal plants in a protected area, natural habitat, or a place that is only a short distance from where the plants naturally grow [4, 20, 41]. Although the populations of many wild species are under heavy pressure because of overexploitation, habitat degradation and invasive species, wild nurseries can provide an effective approach for in situ conservation of medicinal plants that are endemic, endangered, and in-demand [27, 42].

#### Ex situ conservation

Ex situ conservation is not always sharply separated from in situ conservation, but it is an effective complement to it, especially for those overexploited and endangered medicinal plants with slow growth, low abundance, and high susceptibility to replanting diseases [4, 43, 44]. Ex situ conservation aims to cultivate and naturalize threatened species to ensure their continued survival and sometimes to produce large quantities of planting material used in the creation of drugs, and it is often an immediate action taken to sustain medicinal plant resources [45, 46]. Many species of previously wild medicinal plants can not only retain high potency when grown in gardens far away from the habitats where they naturally occur, but can have their reproductive materials selected and stored in seed banks for future replanting [4].

##### Botanic gardens

Botanic gardens play an important role in ex situ conservation [43], and they can maintain the ecosystems to enhance the survival of rare and endangered plant species [38]. Although living collections generally consist of only a few individuals of each species and so are of limited use in terms of genetic conservation [47], botanic gardens have multiple unique features. They involve a wide variety of plant species grown together under common conditions, and often contain taxonomically and ecologically diverse flora [48]. Botanic gardens can play a further role in medicinal plant conservation through the development of propagation and cultivation protocols, as well as undertaking programs of domestication and variety breeding [49].

##### Seed banks

Seed banks offer a better way of storing the genetic diversity of many medicinal plants ex situ than through botanic gardens, and are recommended to help preserve the biological and genetic diversity of wild plant species [50, 51]. The most noteworthy seed bank is the Millennium Seed Bank Project at the Royal Botanic Gardens in Britain [51]. Seed banks allow relatively rapid access to plant samples for the evaluation of their properties, providing helpful information for conserving the remaining natural populations [50, 51]. The challenging tasks of seed banking are how to reintroduce the plant species back into the wild and how to actively assist in the restoration of wild populations [50].

### Cultivation practice

**Fig. 5:**
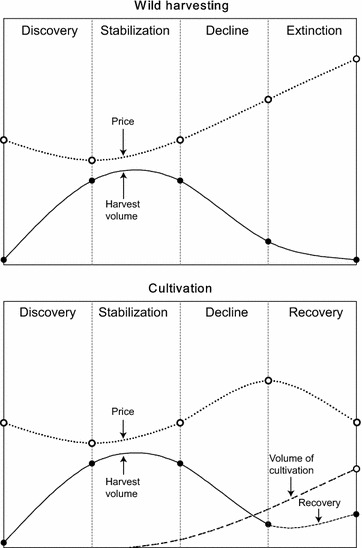
Price and harvest volume variation in the transition from wild-harvesting to cultivation of medicinal plants. As wild resources decline with overharvesting, the price of raw material increases accordingly. Therefore, cultivation becomes economically feasible for price stabilization and resource recovery of medicinal plants. Data sources from Hamilton [4], Larsen and Olsen [13], Schippmann et al. [20], Chan et al. [56], and Schippmann et al. [67]

Although wild-harvested resources of medicinal plants are widely considered more efficacious than those that are cultivated, domestic cultivation is a widely used and generally accepted practice [30, 52, 53]. Cultivation provides the opportunity to use new techniques to solve problems encountered in the production of medicinal plants, such as toxic components, pesticide contamination, low contents of active ingredients, and the misidentification of botanical origin [54]. Cultivation under controlled growth conditions can improve the yields of active compounds, which are almost invariably secondary metabolites, and ensures production stability (Table 3). Cultivation practices are designed to provide optimal levels of water, nutrients, optional additives, and environmental factors including temperature, light and humidity to obtain improved yields of target products [27, 55]. Moreover, increased cultivation contributes to decreases in the harvest volume of medicinal plants, benefits the recovery of their wild resources, and decreases their prices to a more reasonable range [4, 13, 20] (Fig. 5).

**Table 3:** The advantages and disadvantages of wild resource versus cultivated medicinal plant species

Characteristics	Wild resource	Cultivated species
Advantages	It is open access resource without investment	It relieves harvesting pressure on rare and threatened species
	It is natural resource and free from pesticides	It can keep genotypes being standardized or improved
	Wild resource is supposed to be more efficacious	It guarantees continuing supply of raw medicinal materials
		Production volume and price can be stable for longer periods
Disadvantages	Wild resource is becoming scarce and threatened by over-harvesting	It needs substantial investment before and during production
	There exists a risk of adulterations and resource exhaustion	It narrows genetic diversity in gene pool of wild populations
	Uncontrolled harvesting leads to the extinction of ecotype and species	Reintroduced plants can cause genetic pollution of wild resource
	There is a lack of resource inventories and related management practices	Cultivated species may have negative impacts on ecosystems
		There is a lack of successful cultivation techniques for some species

Information from Hamilton [4], Schippmann et al. [20], Liu et al. [27], and Raina et al. [54]

### Good agricultural practices (GAP)

Good agricultural practices (GAP) for medicinal plants have been formulated to regulate production, ensure quality, and facilitate the standardization of herbal drugs [56]. A GAP approach ensures high quality, safe and pollution-free herbal drugs (or crude drugs) by applying available knowledge to address various problems [57]. GAP include comprehensive items, such as the ecological environment of production sites, germplasm, cultivation, collection, and quality aspects of pesticide detection, macroscopic or microscopic authentication, chemical identification of bioactive compounds, and inspection of metal elements [58]. Many countries actively promote the implementation of GAP. For example, Chinese authorities have promoted GAP for the growth of commonly used herbal drugs in regions where those medicinal plants are traditionally cultivated [27, 33].

Organic farming has received increasing attention for its ability to create integrated, humane, and environmentally and economically sustainable production systems for medicinal plants, [59, 60]. The aims of organic farming of medicinal plants include producing material with better quality and high productivity, and ensuring the conservation and sustainable utilization of those plants (Table 4). The defining characteristic of organic farming is the non-use of synthetic fertilizers, pesticides and herbicides, which are not allowed according to many current organic certification standards in Europe and North America [59]. Organic farming is benign to the environment, and relies upon farm-derived renewable resources to maintain biological processes of medicinal plants and ecological balance of habitates [56, 59]. The use of organic fertilizers continuously supplies soil nutrients and improves soil stability, significantly affecting the growth of medicinal plants and the biosynthesis of essential substances. For example, when organic fertilizers were applied, the biomass yield of *Chrysanthemum balsamita* was increased and its essential oil content was high relative to those free from organic fertilizers [61]. Organic farming of medicinal plants is becoming increasingly important in the long-term development and sustainability of medicinal plants [60].

**Table 4:** The characteristics and advantages of organic farming of medicinal plants

Subjects	Characteristics and advantages of organic farming
Medicinal plants	To produce material in optimal quality and sufficient quantity
	To increase growth rate and biomass yield of medicinal plants
	To enhance the biosynthesis of efficacious substances
	To maintain the genetic diversity of medicinal plants
	To protect medicinal plants against pests and disease
Environmental effects	To prohibit the use of synthetic pesticides and fertilizers
	To promote sustainable use and proper care of production systems
	To enhance plant diversity and biotype conservation
	To protect wildlife habitats (e.g. micro-organisms, soil fora and fauna)
	To enhance soil rich in macro and microelements
	To conserve soil properties, fertility, productivity and system stability
	To use organic fertilizers and renewable resources to minimize all forms of pollution
Economic prospects	To increase market opportunity
	To maintain high market price
	To achieve optimal quality and economic returns
	To secure economic growth and social stability

Information from Rigby and Cáceres [59], Macilwain [60] and Suresh [61]

### Sustainable use

For medicinal plants with limited abundance and slow growth, destructive harvesting generally results in resource exhaustion and even species extinction [13, 62]. Therefore, the sustainable use of medicinal plants should be considered, and good harvesting practices must be formulated. Root and whole-plant harvesting is more destructive to medicinal plants (e.g. herbs, shrubs and trees) than collecting their leaves and flowers or buds (Table 5). For herbal drugs made of whole plants or roots, using their leaves as a remedy can be a benign alternative. For example, Wang et al. [63] discovered that extracts from ginseng leaf-stems and roots have similar pharmacological activities, but ginseng leaf-stem has the advantage of being a more sustainable resource.

**Table 5:** Susceptibility of species to overharvesting regarding life forms and plant parts used

Life form	Percent (%)	Leave	Flower/bud	Fruit/seed	Bark	Root	Whole plant
Herb	52	Medium	Medium	High	None	High	High
Shrub	16	Low	Low	Low	High	High	High
Tree	22	Low	Low	Low	High	High	High

Information from Schippmann et al. [67] and Teklehaymanot and Giday [68]

### Prospects

The development of genetic engineering has led to the feasibility of large-scale biosynthesis of natural products, and advancements in tissue culture and fermentation of medicinal plants have opened new avenues for the large-scale and highly efficient production of desirable bioactive compounds. Tissue culture (including plant cell and transgenic hairy root culture) is a promising alternative for the production of rare and high-value secondary metabolites of medical importance [64]. Micropropagation via tissue encapsulation of propagules can not only facilitate storage and transportation, but also promotes higher regeneration rates [62]. When the amounts of normal seeds are insufficient for propagation, synthetic seed technology, defined as artificially encapsulated somatic embryos (or other tissues) could be used for cultivate in vitro or ex vitro, is a feasible alternative [65, 66]. Furthermore, breeding improvements can be carried out using molecular marker-based approaches applied at the genetic level, and the time required for breeding may be significantly shortened [62, 64, 65].

## Conclusion

Despite the existence of various sets of recommendations for the conservation and sustainable use of medicinal plants, only a small portion of these have achieved adequate protection of medicinal plant resources through conventional conservation in natural reserves or botanic gardens.

## Abbreviations

GAP: good agricultural practices
